# Two Complicated Nervous Cases, with Treatment

**Published:** 1902-03

**Authors:** Ernest Vincent

**Affiliations:** Texas


### CLINICAL REPORTS.

### Two Complicated Nervous Cases, with Treatment.

By ERNEST VINCENT, M. D., Texas.

Case I.—A.N.M.C., woman; American, aged 47. First saw this case October 1, 1900. Patient had been under treatment of various physicians for about five years. At time of my first visit, had been unable to walk for about seventeen months. On examination I found partial paralysis, from hips down, alarming cardiac weakness and dropsy of feet and legs, which were swollen to a remarkable size. In addition, the patient was suffering from two chronic leg ulcers, which had resisted all treatment. One was so deep as to partially expose the periosteum. The edges were jagged and the surrounding tissue soft. The two previous physicians in the case had told the husband that nothing further could be done, and to let patient have as much morphia as she wanted. I found that she was not only taking from four to six grains, hypodermatically, daily, but putting morphia in the ulcers to relieve the intensity of the pain. This aggravated the case greatly, increasing the nervous prostration, hysteria and the like.

I prescribed first elaterium and heart tonics which overcame the dropsical conditions. I treated the ulcers with the peroxide of hydrogen and bovinine treatment, and they healed in thirty days. One week after taking charge of the case, I prescribed mercauro, ten drops t.i.d., gradually increasing dose to fifteen drops. When second bottle of mercauro was used, I prescribed arsenauro, watching case closely and gradually pressing dosage until the patient was saturated. At end of eight weeks after commencing treatment the patient could walk with the aid of crutches, which she was soon able to abandon. I discharged her cured, February, 1901, and she is still enjoying good health. When I was satisfied that I had reached the point of saturation, I gradually reduced dosage. I gave as high as twenty-five drops with no ill-effects. A peculiar feature in this case is, that I had no difficulty in breaking up the morphine habit. She never once asked for it. She has since said, "I did not want it. I took it for the pain and your first treatment stopped the pain."

Case II.—M. C., female; American, aged 48 years. First saw this case March, 1900. Patient was apparently insane. I found on inquiry that she had been suffering from nervous prostration for about four years, during the climacteric. She cried constantly, would tear her finger nails with her teeth until blood would flow. Was in the habit of running away from home, threatening suicide and the like. She had been under treatment of several physicians here, who at the time I was called in had informed the husband, a merchant, that her mind was entirely gone and that the only thing to do was to send her to an asylum.

I must confess that the case on first acquaintance looked hopeless and I hesitated a day, before giving the husband my opinion in the case. During these twenty-four hours I watched patient closely and finally encouraged the hope of relief. Having had some success with arsenauro and mercauro, I resolved to put them to a test in this case. I commenced with mercauro, ten drops t.i.d., and before first bottle was used, had the satisfaction of seeing a great change in my patient. I by this time had her completely under control, could reason with her a little and she would obey me when she would no one else. She would come to me at regular times and I would give her the medicine myself. She still had her crying spells, but she ceased biting her nails and lost the wild, haunted expression she had had. Her appetite was remarkable, always eating, and this was a feature in the case that gave me some trouble. Instead of sitting up crying all night, natural short sleeps came to my aid. At the end of a month I used arsenauro, pressing the dosage with great care until full effect. The hysterical spells ceased, appetite became normal, mind much stronger, no more running away from home or threats of suicide. To make a long story short, I discharged case cured in ninety days.

Patient continued well for a year, when I was again called to her. Found her again despondent and mentally upset. Said she was "going to die," could see "death written on the wall," and the like. On inquiry I found that she had been examined two months previously by a physician, who told her it was necessary to treat the womb. He had curetted, and she had been suffering from uterine hemorrhages at intervals ever since. The excessive loss of blood had weakened and frightened her. I ordered absolute rest with good nursing, prescribed arsenauro again, and in a month she was quite well.

